# Circ_0075825 promotes gastric cancer progression via adsorbing miR-432-5p to modulate SOX9

**DOI:** 10.1016/j.clinsp.2022.100018

**Published:** 2022-04-05

**Authors:** He Li, Xiaohua Zhou, Zhuangming Yu, Youjing Tian

**Affiliations:** Department of General Surgery, The First Affiliated Hospital of Hainan Medical University, Hainan, China

**Keywords:** GC, Circ_0075825, miR-432-5p, SOX9

## Abstract

**Methods:**

Circ_0075825 expression in adjacent tissues and GC tissues was evaluated by bioinformatics method and quantitative Reverse Transcription Polymerase Chain Reaction (qRT-PCR). How circ_0075825 regulated GC cell growth, migration, invasion, and apoptosis were investigated by cell counting kit-8 assay, transwell assay and flow cytometry. The targeted interplays among circ_0075825, and miR-432-5p and Sex-Determining Region Y-related high-mobility group box 9 (SOX9) were explored by bioinformatics analysis and luciferase reporter gene assay. The regulatory effects of circ_0075825 and miR-432-5p on SOX9 protein expression were probed by western blot.

**Results:**

Circ_0075825 expression was raised in GC tissues and cell lines. Circ_0075825 overexpression promoted the proliferative, migrative and invasive abilities of GC cells, while inhibiting apoptosis, while depletion of circ_0075825 suppressed the malignant biological behaviors of GC cells. *SOX9* was identified as one of the direct target genes of miR-432-5p, and circ_0075825 repressed the expression of miR-432-5p, to induce the expression of SOX9. Furthermore, miR-432-5p overexpression counteracted the promoting effect of circ_0075825 on the malignancy of GC cells.

**Conclusion:**

Circ_0075825 promotes GC progression via sponging miR-432-5p to regulate SOX9 expression level, and it may be a novel therapeutic target for treating GC.

## Highlights

• Circ_0075825 is highly expressed in gastric cancer tissues.
• Circ_0075825 promotes the malignant biological behaviors of gastric cancer cells.
• Circ_0075825 functions as a molecular sponge to repress miR-432-5p and up-regulate SOX9.

## Introduction

The incidence of Gastric Cancer (GC) ranks fifth among human malignancies, and GC ranks third in the causes of cancer-related deaths.^1^ The prognosis of GC patients with distant metastasis or recurrence after surgery is extremely poor; and there are few treatment strategies to prolong their survival time.^2^, ^3^, ^4^ Therefore, it is pivotal to delve into the mechanism pertinent to GC progression to provide clues to improve the diagnosis and treatment of GC.

In recent years, accumulating evidence has supported that non-coding RNA (ncRNA) regulates various biological activities.^5^ Circular RNA (circRNA), an emerging category of ncRNA, is characterized by a covalently closed loop structure with neither 5′ to 3′ polarity nor polyadenylated tail.^6^ CircRNA is abnormally expressed in many cancers, and the dysregulation of circRNA is associated with the malignancy of cancer cells.^7^ For example, circ-HuR is underexpressed in GC tissues and cell lines, and mechanically, circ-HuR interferes with CCHC-type zinc finger nucleic acid-binding protein, and thus its binding to HuR promoters is restrained, and as a result, GC cell growth and metastasis are repressed.^8^ As reported, as a member of circRNA family, circ_0075825 is in high expression in the peripheral blood of GC patients,^9^ but its role and mechanism in GC deserve further investigation. In recent years, in the field of cancer research, more and more studies report that circRNAs competitively bind with microRNA (miRNA) to regulate downstream target 'genes' expression.^10^, ^11^, ^12^, ^13^ For example, circRNA_100290 serves as a Competing Endogenous RNA (ceRNA) and modulate CDK6 expression via adsorbing miR-29b family members, and thus the progression of oral squamous cell carcinomas is accelerated.^11^

In this study, the authors identified the differentially expressed circRNAs with the microarray data from Gene Expression Omnibus (GEO) database, and it was revealed that circ_0075825 expression was up-regulated in GC tissues and cell lines. Functionally and mechanistically, circ_0075825 can increase SOX9 expression via adsorbing miR-432-5p, thus facilitating the progression of GC.

## Materials and methods

### Collection of tissue specimens

Fresh GC tissues and adjacent tissue samples (> 3 cm from the tumor margin) from fifty patients diagnosed with GC were collected from May 2017 to April 2019 in the First Affiliated Hospital of Hainan Medical University. The tissue samples were stored at -80 °C after radical surgery. The tissues samples were confirmed as GC tissues or cancer cell-free tissue samples by two experienced pathologists. All of the enrolled patients 'didn't receive any anti-cancer treatments before the tissue sample collection. Only patients with gastric adenocarcinoma were included, and the patients with gastric lymphoma, gastrointestinal stromal tumors, and other malignancies were excluded. All patients provided written informed consent. This work was endorsed by the Ethics Committee of the First Affiliated Hospital of Hainan Medical University.

### Ethics approval and consent to participate

This study was approved by the Institutional Ethics Committee of The First Affiliated Hospital of Hainan Medical University (Study protocol number: HMU-2019-008), and informed consent was obtained from all patients before the research.

### Bioinformatics analysis

The dataset GSE93541 was available from the GEO database (https://www.ncbi.nlm.nih.gov/gds) and analyzed by GEO2R tool. GEO2R was also used to generate the volcano map and heat map. Adjusted *p* < 0.05, False Discovery Rate (FDR) < 0.05, and | log_2_ (Fold Change) | > 2 were set as the thresholds for screening the differentially expressed circRNAs in GC tissues. circinteractome database (https://circinteractome.irp.nia.nih.gov/) was used to predict the binding site between circ_0075825 and miR-432-5p, and StarBase database (starbase.sysu.edu.cn/) was used to predict the binding site between miR-432-5p and the '3′UTR of SOX9.

### Cell culture

GC cell lines (AGS, NUGC4, MKN74 and BGC-803) and normal human gastric epithelial cell line GES-1 were from American Type Culture Collection (Manassas, VA, USA). These cells were cultured with ' 'Dulbecco's Modified Eagle medium (DMEM) (Gibco, Carlsbad, CA, USA) with 10% fetal bovine serum (FBS, Gibco), 100 U/mL penicillin, and 100 μg/mL streptomycin (Gibco) in 5% CO_2_ at 37 °C.

### Cell transfection

The overexpression vectors of SOX9 and circ_0075825 were constructed with pcDNA3.1 vector. The overexpression vector, empty vector (used as the control for the overexpression vector), Small Interfering RNAs (siRNAs) for circ_0075825 (si-circ_0075825-1: 5’-GTGGCTAATGGTGTGAATCAT-3’; si-circ_0075825-2: 5’-AGGAAGTGGCTAATGGTGTGA-3’), control siRNA, miR-432-5p mimics, miR-432-5p inhibitor and miRNA negative control (miR-control) were available from RiboBio Co., Ltd. (Guangzhou, China). Subsequently, they were transfected into the cells by Lipofectamine^TM^ 2000 (Invitrogen, Carlsbad, CA, USA) as instructions.

### Quantitative reverse transcription PCR (qRT-PCR)

Total RNA was isolated from the GC tissues and cells by TRIzol reagent (Invitrogen). The RNA was reversely transcribed into cDNA by a PrimeScript^TM^ RT reagent Kit (TaKaRa, Dalian, China). qRT-PCR was performed with an SYBR® Premix Ex Taq^TM^ II Kit (TaKaRa) in ABI7500 System (Applied Biosystems, Foster City, CA, USA), with Glyceraldehyde 3-Phosphate Dehydrogenase (GAPDH) and U6 as the internal references. The 2^−ΔΔCt^ method was used to quantify the relative expression of each target gene. Primer sequences are listed in Table 1.

**Table 1:** Sequences used for qRT-PCR.

**Name**	**Primer sequences**
circ_0075825	Forward: 5′-GGATGGCTGTTCTCCATTGT-3′
	Reverse: 5′-TATACATGCACGCCCTCAAA-3′
miR-432-5p	Forward:5′-AACGAGACGACGACAGAC-3′
	Reverse:5′-CTTGGAGTAGGTCATTGGGT-3′
U6	Forward:5′-AACGAGACGACGACAGAC-3′
	Reverse:5′-GCAAATTCGTGAAGCGTTCCATA-3′
SOX9	Forward: 5′-CAAGAAGGACCACCCGGATT-3′
	Reverse: 5′-AAGATGGCGTTGGGGGAGAT-3′
CTNNB1	Forward: 5′-AAAGCGGCTGTTAGTCACTGG-3′
	Reverse: 5′-CGAGTCATTGCATACTGTCCAT-3′
COL10A1	Forward: 5′-ATGCTGCCACAAATACCCTTT-3′
	Reverse: 5′-GGTAGTGGGCCTTTTATGCCT-3′
GAPDH	Forward:5′-GACTCATGACCACAGTCCATGC-3′
	Reverse:5′-AGAGGCAGGGATGATGTTCTG-3′

### Cell proliferation assay

Cell Counting Kit-8 (CCK-8) (Meilunbio, Shanghai, China) was adopted to assess GC 'cells' proliferative ability. NUGC4 and BGC-803 cells were cultured in 96-well plates (1 × 10^3^ cells/well) for 24 h, 48 h, 72 h and 96 h, respectively. Then the cells were incubated with 10 µL of CCK-8 solution for 4 h at 37 °C. The absorbance value of OD_450 nm_ was detected with a microplate reader.

### Transwell assay

For migration assay, 1 × 10^5^ transfected cells were resuspended in serum-free medium and plated into the upper chamber of transwell inserts (8 µm; Corning Inc., Corning, NY, USA). Next, the inserts were then positioned into the 24-well plate containing 500 µL of DMEM with 10% FBS in each well. Cells were cultured at 307 °C for 24 h, and the remaining cells on the upper surface of the membrane were immediately wiped off with a cotton swab, and those cells on the below surface of the membrane were fixed with 95% ethanol for 20 min and stained with 0.1% crystal violet for 10 min. Ultimately, the stained cells were counted. For invasion assays, the membrane was pre-coated with diluted Matrigel, and the following procedures were the same with the migration assay.

### Flow cytometry assay

The apoptosis of the transfected cells was evaluated with the Annexin V-FITC apoptosis detection kit (Invitrogen). NUGC4 and BGC-803 cells were rinsed in cold PBS. The transfected cells were resuspended in 100 μL of binding buffer (1 × 10^6^ cells/mL). Then the resuspended cells were stained with 10 μL of Annexin V-FITC staining solution and 5 μL of PI staining solution at ambient temperature for 20 min in darkness. Cells apoptosis was subsequently analyzed by a flow cytometer (BD Biosciences, San Jose, CA, USA).

### Western blot

Total proteins of the cells were extracted by RIPA buffer (Biosharp, Hefei, China), with concentrations quantified by a BCA protein assay kit (Beyotime, Haimen, China). Proteins were separated by sodium dodecyl sulfate-polyacrylamide gel electrophoresis and transferred onto polyvinylidene fluoride membranes (Millipore, Bedford, MA, USA), which was then blocked with 5% skimmed milk for 1h at room temperature, and followingly incubated with the primary antibodies specific for SOX9 (1:1000, ab185966, Abcam, Shanghai, China) and GAPDH (1:5000, ab8245, Abcam) at 4 °C overnight. Subsequently, the membranes were incubated with Horseradish Peroxidase (HRP) conjugated secondary antibodies (Abcam) at ambient temperature for 1h. The protein bands were developed by an Amersham Imager 600 (GEHealthcare, Chicago, IL, USA) with chemiluminescence reagent (Beyotime).

### Dual-Luciferase reporter gene assay

Firstly, circ_0075825 or SOX9 3′UTR sequence containing Wide-Type (WT) or Mutant-Type (MUT) binding site for miR-432-5p were inserted into pGL3 luciferase reporter vector (Promega, Madison, WI, USA) to establish circ_0075825-WT, circ_0075825-MUT, and SOX9-3′UTR-WT, SOX9-3′UTR-MUT reporter vectors. Secondly, reporter plasmids were, respectively transfected into HEK-293T cells with miR-432-5p mimics or control miRNA. 48h later, the luciferase activity was determined by the dual-luciferase reporter assay system (Promega). The relative firefly luciferase activity was normalized to the Renilla luciferase activity.

### Statistical analysis

SPSS version 20.0 statistical software (IBM, Armonk, NY, USA) was employed for statistical analysis. All data were expressed as mean ± standard deviation. Whether the data were normally distributed or not was tested with the Kolmogorov-Smirnov test. Paired *t*-test or unpaired *t*-test was used for making comparisons between two groups. One-way ANOVA with Tukey's post hoc test was adopted for making the comparisons among multiple groups. ' 'Spearman's correlation analysis was conducted to evaluate the interrelation between circ_0075825 expression and miR-432-5p expression or SOX9 expression in GC tissues, respectively. Chi-Square test was used to analyze the association between circ_0075825 expression level and the 'patients' pathological characteristics; *p* < 0.05 was considered statistically significant.

## Results

### Circ_0075825 expression is raised in GC tissues

**Fig. 1:**
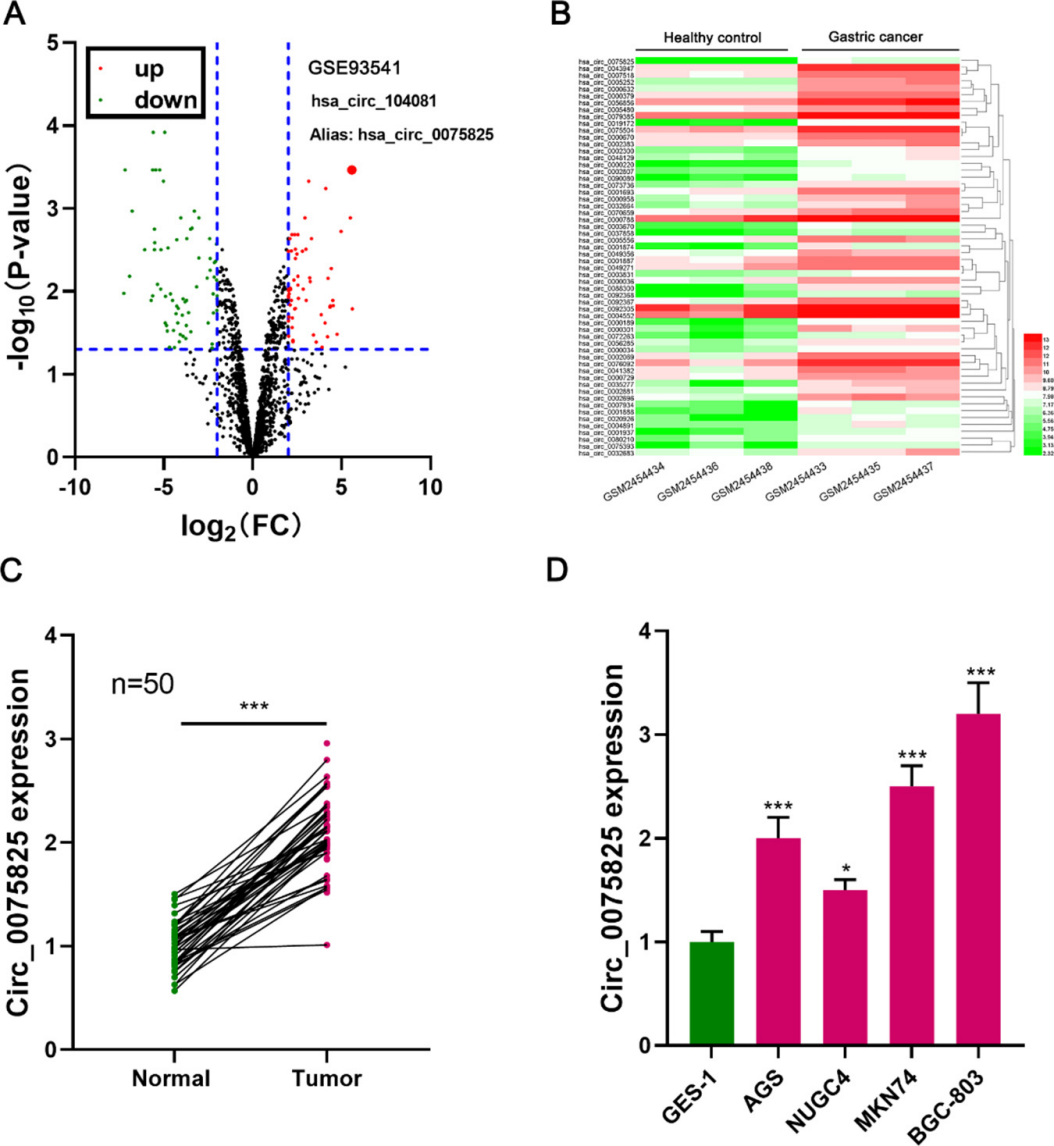
Circ_0075825 is highly expressed in GC. (A) The volcano plot showed the up-regulation and down-regulation of circRNAs in the plasma of GC patients vs. healthy controls. Up-regulated circRNAs were indicated by """"red"""", and down-regulated circRNAs were indicated by """"green"""", and no significant difference was denoted by """"black"""". (B) Heat map showed some representative dysregulated circRNAs in GSE93541. (C) qRT-PCR assay was used to detect the expression of circ_0075825 in human GC tissues (*n* = 50) and adjacent tissues (*n* = 50). (D) qRT-PCR assay was used to detect the expression of circ_0075825 in normal Gastric Epithelial cells (GES-1) and GC cell lines (AGS, NUGC4, MKN74, and BGC-803). FC, Fold Change; *p < 0.05, *** *p* < 0.001

GSE93541, containing circRNA expression profile data of three plasma samples of GC patients and three healthy controls, was available from the GEO database. The differentially expressed circRNAs were identified (Fig. 1 A,B). Circ_0075825 was the circRNA with the most significant up-regulation in GC patients (log_2_ fold change = 5.3) (Fig. 1 A,B). Next, circ_0075825 expression in GC tissues was detected by qRT-PCR, and it was revealed that the expression of circ_0075825 was dramatically higher than that in adjacent tissues (fold change = 2.2) (Fig. 1C). Additionally, the 50 patients were averagely divided into the high expression group and low expression group (25 vs. 25) according to the expression level of circ_0075825 in tumor tissue; high expression of circ_0075825 was associated with higher T-stage and lymphatic metastasis of the patients with GC (Table 2). This suggested that circ_0075825 could probably be associated with GC progression. Also, circ_0075825 expression in GC cell lines (AGS, NUGC4, MKN74, and BGC-803) was markedly higher than that in the GES-1 cell line (Fig. 1D). Among the four GC cell lines, the expression level of circ_0075825 was highest in BGC-803 cells and lowest in the NUGC4 cells. So these two cell lines were used in subsequent experiments.

**Table 2:** Association between circ_0075825 expression and clinicopathological characteristics of GC patients.

**Characteristics**	**All case (n** = **50)**	**Circ_0075825 expression**		**χ^2^**	**p-value**
		**High**	**Low**		
Gender					
Male	39	20	19	0.117	0.733
Female	11	5	6		
Age(years)					
≤ 45	16	10	6	1.471	0.225
> 45	34	15	19		
Family history of cancer					
Yes	29	13	16	0.739	0.390
No	21	12	9		
T-stage					
I, II	28	8	20	11.69	< 0.001^a^
III, IV	22	17	5		
Tumor size					
< 3 cm	32	13	19	3.125	0.077
≥ 3 cm	18	12	6		
Lymphatic metastasis					
No	19	6	13	4.160	0.041^a^
Yes	31	19	12		
Infection with helicobacter pylori					
Yes	14	6	8	0.397	0.529
No	36	19	17		

a *p* < 0.05.

### Circ_0075825 promotes GC cell multiplication, migration and invasion, while suppressing the apoptosis

**Fig. 2:**
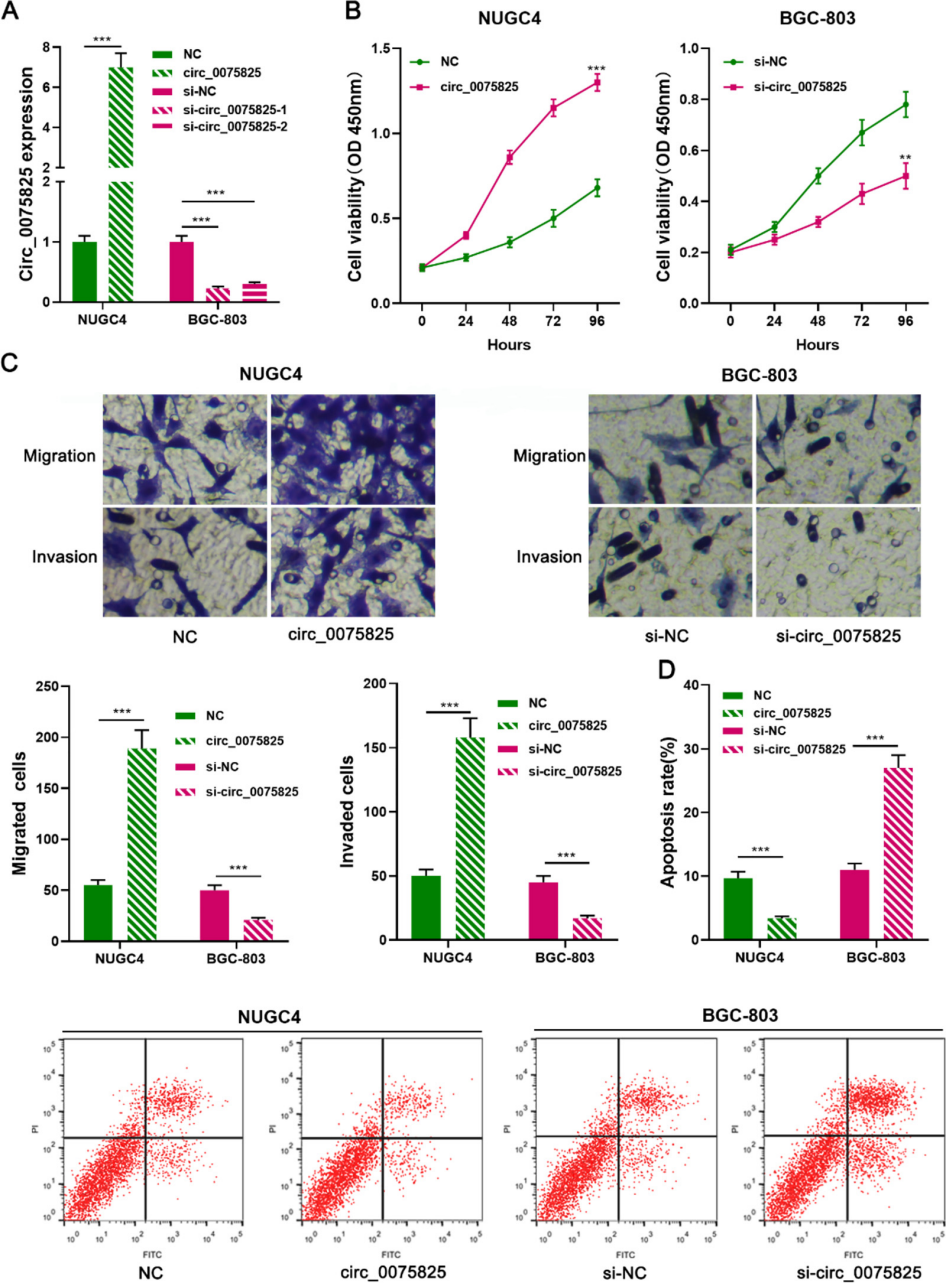
Circ_0075825 promotes GC cells proliferation, migration, and invasion while inhibiting apoptosis. (A) qRT-PCR assay was employed to confirm that the circ_0075825 overexpression and knockdown cell models were constructed successfully. (B) CCK8 assay was used to detect the proliferation of GC cells. (C) Transwell assay was used to detect the migration and invasion of GC cells. (D) Flow cytometry assay was used to detect the apoptosis rate of GC cells. si, siRNA; NC, Negative Control. **p < 0.01, and ***p < 0.001

To expound how circ_0075825 impacts GC progression, the authors successfully overexpressed or silenced circ_0075825 expression in NUGC4 and BGC-803 cells, respectively (Fig. 2A). Given that si-circ_0075825-1 was more effective than si-circ_0075825-2 in BGC-803 cells, so si-circ_0075825-1 was selected for the following experiments. CCK-8 and transwell assay showed that circ_0075825 could significantly strengthen the viability, migration, and invasion of NUGC4 cells (Fig. 2 B,C). Flow cytometry assay showed that overexpression of circ_0075825 repressed apoptosis (Fig. 2D). However, knocking down circ_0075825 significantly suppressed the malignant biological behaviors of BGC-803 cells and promoted apoptosis (Fig. 2 B,D). Those results indicated that circ_0075825 promoted the progression of GC.

### Circ_0075825 directly interacts with miR-432-5p

**Fig. 3:**
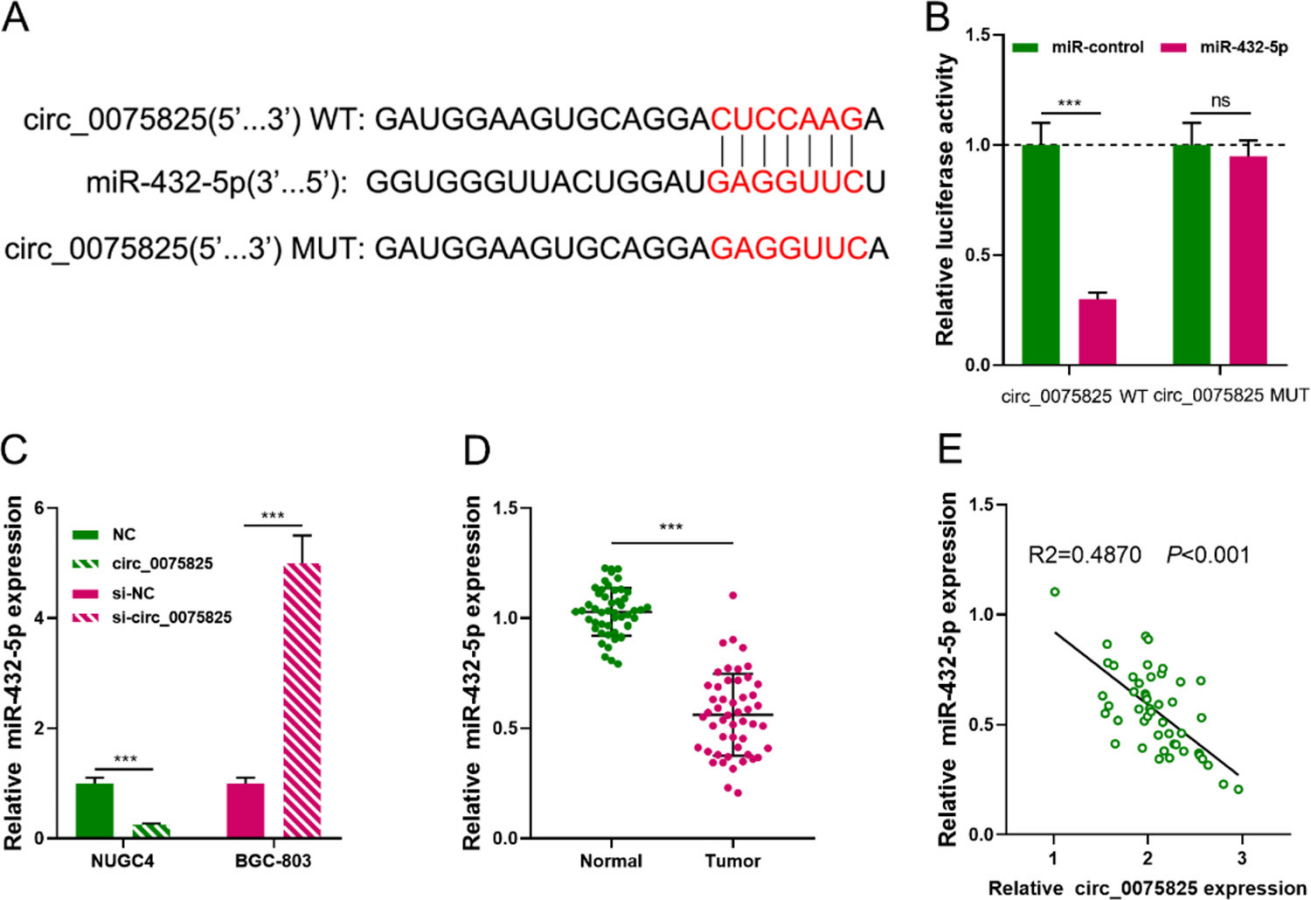
Circ_0075825 directly interacts with miR-432-5p. (A) Circinteractome online bioinformatics tool was used to predict the binding site between circ_0075825 and miR-432-5p. (B) Dual-luciferase reporter assay showed that circ_0075825 adsorbed miR-432-5p. (C) qRT-PCR assay was used to detect the expression of miR-432-5p in GC cells after transfection with circ_0075825 or si-circ_0075825. (D) The expression of miR-432-5p in GC tissues (*n* = 50) and adjacent tissues (*n* = 50) was detected by qRT-PCR assay. (E) ' 'Spearman's correlation analysis was used to analyzing the correlation between circ_0075825 expression and miR-432-5p expression in GC tissues (*n* = 50). WT, Wild Type; MUT, Mutant Type; si, siRNA; NC, Negative Control; ns, not significant; ***p < 0.001.

Next, circinteractome database was searched to predict the potential miRNA targets of circ_0075825, and a total of 20 candidates were obtained, including miR-1184, miR-1205, miR-1208, miR-1248, miR-1252, miR-1253, miR-1265, miR-1287, miR-1289, miR-1304, miR-377, miR-432, miR-494, miR-558, miR-561, miR-576-3p, miR-580, miR-607, miR-625 and miR-936. Among them, miR-432-5p, which had the highest """context+ score percentile""" (99%), attracted our attention (Fig. 3A). Dual-luciferase reporter assay highlighted that miR-432-5p suppressed the luciferase activity of the circ_0075825-WT group but not significantly changed that of the circ_0075825-MUT group (Fig. 3B). qRT-PCR showed that overexpression of circ_0075825 inhibited miR-432-5p expression while knocking down circ_0075825 worked oppositely (Fig. 3C). Besides, miR-432-5p expression in GC tissues was greatly lower than those in adjacent tissues (Fig. 3D). Furthermore, Spearman's correlation analysis implied that circ_0075825 expression negatively correlated with miR-432-5p expression in GC tissues (Fig. 3E). So it was concluded that miR-432-5p was a target of circ_ 0075825.

### SOX9 is a direct target of miR-432-5p

**Fig. 4:**
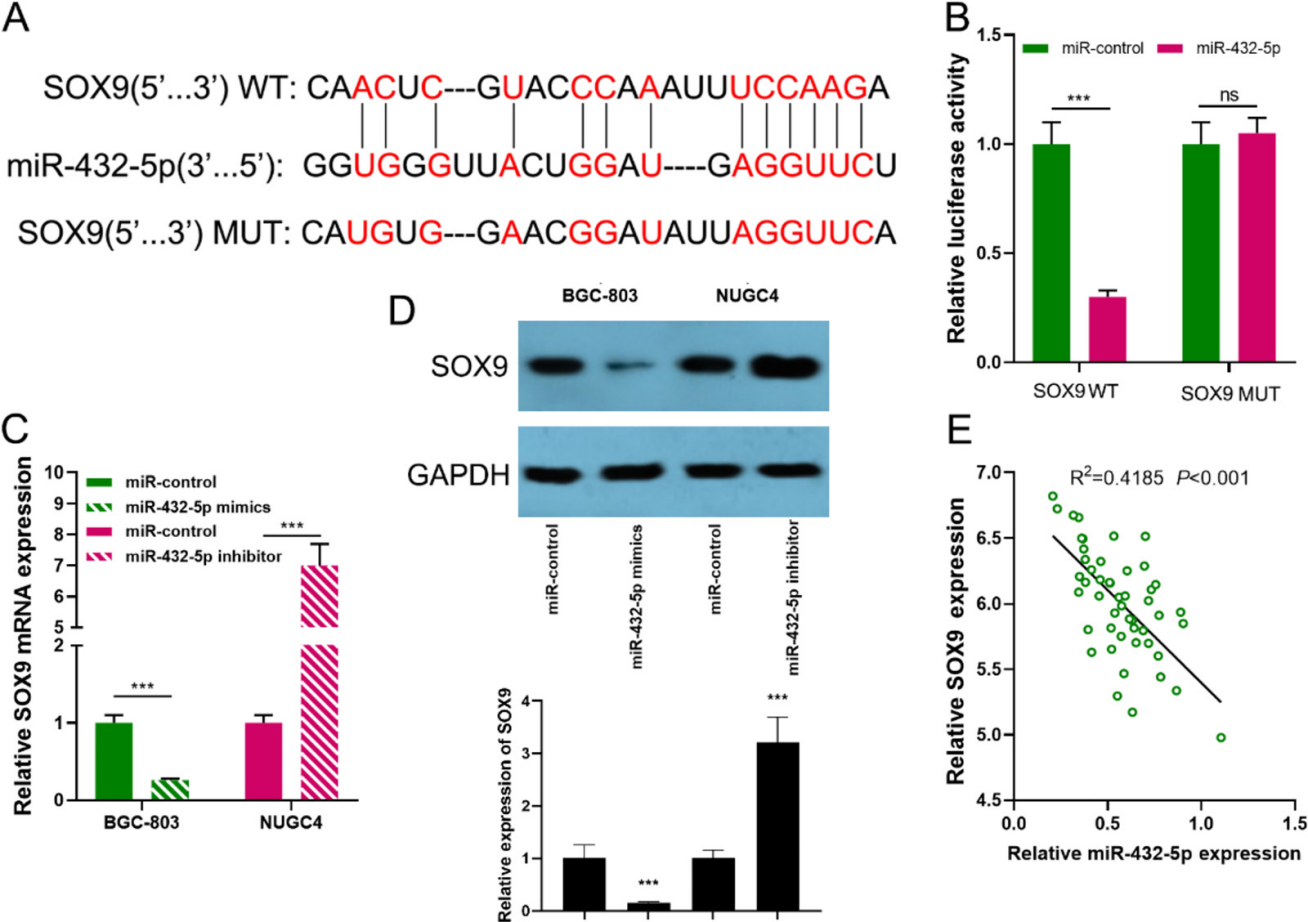
SOX9 is a direct target of miR-432-5p. (A) StarBase database was used to predict the binding site between miR-432-5p and the '3′UTR of SOX9. (B) A dual-luciferase reporter assay was used to confirm the interaction between miR-432-5p and the '3′UTR of SOX9. (C-D) qRT-PCR and western blot assays were used to detect the expression of SOX9 after miR-432-5p was overexpressed or inhibited. (E) ' 'Spearman's correlation analysis was used to analyze the correlation between the expression levels of SOX9 and miR-432-5p in GC tissues (*n* = 50). WT, Wild Type; MUT, Mutant Type; ns, not significant; ***p < 0.001.

Through the StarBase database, a binding site in the SOX9 3′-UTR with miR-432-5p was predicted (Fig. 4A). Dual-luciferase reporter assay highlighted that the transfection of miR-432-5p mimics could markedly inhibit the luciferase activity of SOX9-WT group, but that of SOX9-MUT group was not significantly impacted (Fig. 4B). In qRT-PCR and western blot assays, it was revealed that the transfection of miR-432-5p mimics demonstrably curbed SOX9 expression, while transfection of miR-432-5p inhibitors increased SOX9 expression (Fig. 4 C-D). Furthermore, Spearman's correlation analysis revealed that miR-432-5p expression was negatively correlated with SOX9 expression in GC tissues (Fig. 4E).

### Circ_0075825 accelerates GC progression via modulating miR-432-5p/SOX9

**Fig. 5:**
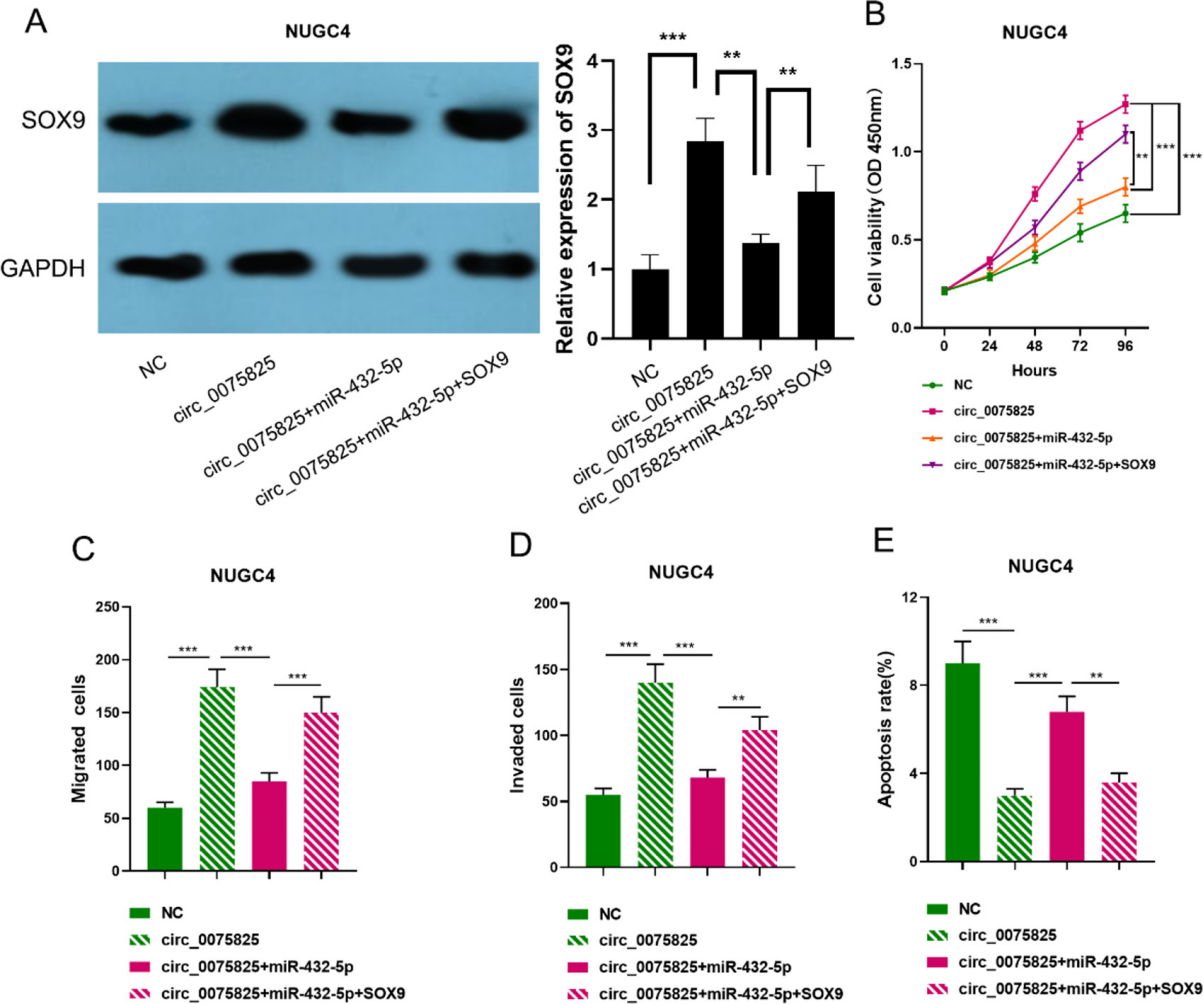
Circ_0075825 promotes malignant phenotypes of GC cells by regulating miR-432-5p / SOX9 axis. (A) NUGC4 cells with circ_0075825 overexpression were transfected with miR-432-5p mimics and SOX9 overexpression vector, and the protein expression of SOX9 in GC cells was detected by western blot assay. (B) CCK-8 assay was used to detect the proliferation of NUGC4 cells after the transfection. (C-D) Transwell assay was used to detect the migration and invasion of NUGC4 cells after the transfection. (E) Flow cytometry assay was used to detect the apoptosis of NUGC4 cells after the transfection. **p < 0.01, and ***p < 0.001.

To delve into whether circ_0075825 functioned via modulating miR-432-5p/SOX9 pathway, the authors conducted rescue assays with NUGC4 cells transfected with circ_0075825 overexpression vector, circ_0075825+ miR-432-5p mimics or circ_0075825+miR-432-5p mimics+SOX9 overexpression vector. Western blot assay has shown that the expression of SOX9 was significantly increased due to circ_0075825 overexpression, while the effect was markedly reversed by co-transfection with miR-432-5p mimics (Fig. 5A). Additionally, circ_0075825 overexpression in NUGC4 cells could induce the expression levels of β-catenin (CTNNB1) and collagen, type X, alpha 1 (COL10A1), two target genes of SOX9; miR-432-5p restoration could partly reverse the change of CTNNB1 and COL10A1 (Supplementary Table 1). These data suggested that circ_0075825/miR-432-5p axis also regulated the downstream pathways of SOX9. CCK-8 assay, transwell assay, and flow cytometry assay showed that circ_0075825 overexpression expedited the multiplication, migration, and invasion and inhibited the apoptosis of NUGC4 cells, and co-transfection with miR-432-5p mimics suppressed GC cell multiplication, migration and invasion, and promoted apoptosis, while SOX9 overexpression worked oppositely (Fig. 5 B–E). These findings showed that circ_0075825 and SOX9 served as cancer promoters, while miR-432-5p was a tumor suppressor to GC. Circ_0075825 might sponge miR-432-5p to modulate SOX9 expression and promote the malignancy of GC cells.

## Discussion

Nowadays, circRNAs are no longer considered as the junk-products in pre-mRNA splicing.^12^^,^^13^ Unlike linear RNA, circRNAs have special covalently closed-loop structures, which are evolutionarily conserved and stable. CircRNA is a promising biomarker in some diseases due to its sequence conservation, high abundance and tissue specificity.^14^ Many biological functions of circRNAs have been unveiled in recent years, for example, acting as scaffolding in the assembly of protein complexes, modulating parental 'genes' expression and alternative splicing, and RNA-protein interactions, and serving as miRNA sponges.^13^^,^^15^^,^^16^ CircRNA, reportedly, is implicated in regulating tumor progression.^17^ For example, circ_100395 expression is negatively interrelated with TNM staging and metastasis of lung cancer, and circ_100395 overexpression significantly represses the malignant biological behaviors of lung cancer cells.^18^ In GC, circ-KIAA1244 is lowly expressed in tumor tissues; circ-KIAA1244 is negatively correlated with TNM staging and lymphatic metastasis, and the low expression of circ-KIAA1244 predicts the shorter survival time of GC patients.^19^ Besides, circRNA_102231_expression in lung adenocarcinoma is significantly up-regulated, which is associated with advanced TNM stage, lymph node metastasis, and poor prognosis.^20^ A previous study reports that the level of circ_0075825 is higher in the plasma of GC patients.^9^ In this work, it was revealed that circ_0075825 was also up-regulated in GC tissues, and it promoted the malignant biological behaviors of GC cells. In the present study, for the first time, reports that circ_0075825 had oncogenic properties in GC cells.

It is well known that miRNA dysregulation contributes to the progression of many malignancies. For example, miR-194 inhibits SUFU and activates Wnt signaling, and thus the GC cell growth and migration are accelerated.^21^ miR-4317 inhibits the multiplication of GC cells via targeting ZNF322.^22^ MiR-432-5p is involved in regulating the progression of glioma, liver cancer, and bladder cancer.^23^, ^24^, ^25^ Notably, circRNA can adsorb miRNA to repress the biological function of miRNA.^10^, ^11^, ^12^, ^13^^,^^26^ In this work, it was revealed that miR-432-5p was underexpressed in GC tissues, and circ_0075825 acted as an endogenous sponge for miR-432-5p to negatively regulate its expression. Similar to its role in other malignancies, miR-432-5p also exerted tumor-suppressive functions in GC cells, counteracting the biological effects of circ_0075825.

SOX9 is an important transcription factor pertinent to stemness, differentiation, and progenitor development.^27^ Besides, SOX9 protein regulates various pathways which are associated with tumor initiation, growth, metastasis, and chemoresistance.^28^ Also, SOX9 is overexpressed in many cancers, such as breast cancer, bladder cancer, and prostate cancer.^29^^,^^30^ Importantly, SOX9 promotes the progression of GC, and its high expression implies a poor prognosis.^31^^,^^32^ Mechanistically, SOX9 interacts with β-catenin to regulate the activity of wnt signaling, and SOX9 also induces the expression of COL10A1 to facilitate the epithelial-mesenchymal transition of GC cells.^31^^,^^32^ In this study, it was demonstrated that SOX9 was a target gene of miR-432-5p, and the expression of SOX9 was positively regulated by circ_0075825. Additionally, circ_0075825 / miR-432-5p axis also regulated the expression of β-catenin and COL10A1 in GC cells. These data suggest that there is a ceRNA network consisting of circ_0075825, miR-432-5p, and SOX9 in GC progression.

In conclusion, circ_0075825 expression is raised in GC tissues and cell lines, and it promotes GC cell proliferative, migrative, and invasive abilities and restrains apoptosis via miR-432-5p / SOX9 axis, implying that circ_0075825 may be a prospective target for treating GC.

## CRediT authorship contribution statement

**He Li:** Visualization, Writing – original draft, Writing – review & editing. **Xiaohua Zhou:** Visualization, Writing – original draft, Writing – review & editing. **Zhuangming Yu:** Methodology, Conceptualization, Visualization, Writing – review & editing. **Youjing Tian:** Formal analysis, Writing – original draft, Writing – original draft, Writing – review & editing.

## Conflicts of Interest

The authors declare no conflicts of interest.

## Appendix Supplementary materials

Image, application 1
