# Supplementary material for: Circ_0075825 promotes gastric cancer progression via adsorbing miR-432-5p to modulate SOX9
Source: Clinics (Sao Paulo). 2022 Apr 5;77:100018. doi: 10.1016/j.clinsp.2022.100018 (PMC8989707; doi:10.1016/j.clinsp.2022.100018)
Supplement: Supplementary file 1 [file mmc1.pdf]

CLINICS-2021-3021_Supplementary Material

Supplementary Table 1 The regulatory effects of circ_0075825, miR-432-5p and SOX9 on the expression level of downstream genes of SOX9**.**

Gene symbol	Control	Circ_0075825	Circ_0075825 + miR-432-5p	Circ_0075825 + miR-432-5p + SOX9
COL10A1	1	4.52	1.44	6.37
CTNNB1	1	6.22	2.55	5.64

The numbers presented the fold change of the expression of the genes compared with that in the control group.
